# K^+^-Responsive Crown Ether-Based Amphiphilic Copolymer: Synthesis and Application in the Release of Drugs and Au Nanoparticles

**DOI:** 10.3390/polym14030406

**Published:** 2022-01-20

**Authors:** Xiao Wang, Xianghong Zheng, Xinyu Liu, Birong Zeng, Yiting Xu, Conghui Yuan, Lizong Dai

**Affiliations:** 1 Department of Materials Science and Engineering, College of Materials, Xiamen University, Xiamen 361005, China; wangxiao_@hust.edu.cn (X.W.); 20720191150089@stu.xmu.edu.cn (X.Z.); lxy212@xmu.edu.cn (X.L.); xyting@xmu.edu.cn (Y.X.); yuanch@xmu.edu.cn (C.Y.); 2 Fujian Provincial Key Laboratory of Fire Retardant Materials, Xiamen 361005, China

**Keywords:** crown ether, amphiphilic copolymers, K^+^-triggered, Au NPs, disassembly

## Abstract

Due to unique chelating and macrocyclic effects, crown ether compounds exhibit wide application prospects. They could be introduced into amphiphilic copolymers to provide new trigger mode for drug delivery. In this work, new amphiphilic random polymers of poly(lipoic acid-methacrylate-co-poly(ethylene glycol) methyl ether methacrylate-co-*N*-isopropylacrylamide-co-benzo-18-crown-6-methacrylamide (abbrev. PLENB) containing a crown ether ring and disulphide bond were synthesized via RAFT polymerization. Using the solvent evaporation method, the PLENB micelles were formed and then used to load substances, such as doxorubicin hydrochloride (DOX) and gold nanoparticles. The results showed that PLENB exhibited a variety of lowest critical solution temperature (LCST) in response to the presence of different ions, such as K^+^, Na^+^ and Mg^2+^. In particular, the addition of 150 mM K^+^ increased the LCST of PLENB from 31 to 37 °C and induced the release of DOX from the PLENB@DOX assemblies with a release rate of 99.84% within 12 h under 37 °C. However, Na^+^ and Mg^2+^ ions could not initiate the same response. Furthermore, K^+^ ions drove the disassembly of gold aggregates from the PLENB-SH@Au assemblies to achieve the transport of Au NPs, which is helpful to construct a K^+^-triggered carrier system.

## 1. Introduction

Many kinds of materials with different morphologies have been studied to promote the development of drug delivery systems. For example, hydrogels are used for drug delivery biomaterial scaffolds due to their three-dimensional porous network, flexibility and high-water content [1]. Nanofibers are used for drug vehicles because of their high surface-area-to-volume ratio associated with great drug encapsulation ability, high drug loading and stability [2,3].

Various nanoparticles are popular for their high surface area, safety and efficacy of encapsulation [4,5,6]. Commonly the aim of drug delivery systems is to maximize the therapeutic activity while minimizing the negative side effects; therefore, the drug leakage and precise targeting are the priority factors. Among of these materials, amphiphilic copolymers have been extensively studied due to their excellent properties, such as the effective prevention of drug leakage [7,8].

It is well known that amphiphilic polymers can form micelles in aqueous solutions and the corresponding composition, morphology and size are easy to control. As a drug carrier, amphiphilic polymers can release drugs at target sites according to different environmental stimuli, such as pH gradients [9], redox potential difference [10,11], ion concentration change [12] etc. Thus far, researchers have made many efforts to broaden the synthesis of new multifunctional copolymers and the development of their applications.

Crown ethers and host-guest chemistry have shown unique advantages in the molecular recognition [13,14], drug design and biological simulation fields [15,16,17]. The crown ether-based supramolecular polymers and gels have received increasing attention due to their special selectivity and coordination to alkali metals and alkaline earth metals [18,19,20,21]. In the previous literature, ion-recognizable smart materials composed of poly(*N*-isopropylacrylamide-co-benzo-18-crown [6]-acrylamide) (abbrev. poly(NIPAM-co-B18C6Am)) copolymer, which exhibited both thermo-responsive and ion-recognition properties, have been developed, such as linear-grafted gating membranes, hydrogels, microspheres and microcapsules [22,23,24,25].

For example, a molecular-recognition microcapsule composed of a core-shell porous membrane and linear grafted poly(NIPAM-co-B18C6Am) chains in the pores acting as molecular-recognition gates was designed for environmental stimuli-responsive controlled release. The results showed that the release of the solute vitamin B_12_ from the prepared microcapsules was significantly sensitive to the presence of Ba^2+^ ions in the environmental solution [26]. Consequently, it is of great importance to fabricate responsive polymers by combining crown ether units with various functional monomers.

Lipoic acid (LA) is a well-known antioxidant with cyclic disulphide, which can be reduced to dihydrolipoic acid in cells [27,28] and be applied in curing many diseases [29,30]. Some organic compounds containing LA and crown ether unit have been successfully synthesized and used in the protection of gold nanoparticles [31] or other application [32]. However, to the best of our knowledge, the responsive copolymer constructed by both crown ether and lipoic acid moiety for the delivery of drug molecules or nanoparticles under external stimuli has still not been reported.

The objective of this study is to fabricate a new amphiphilic copolymer consisting of multiple functional groups and investigate the self-assembly behaviours, ion-responsive characteristics and the release of small molecular drug and disassembly of gold nanoparticle assemblies under external stimuli. First, we prepared benzo-18-crown-6-methacrylamide monomer B18C6Am and lipoic acid-methacrylate monomer LAMA. Secondly, four monomers, including LAMA, poly(ethylene glycol) methyl ether methacrylate (PEGMA), *N*-isopropylacrylamide (NIPAM) and B18C6Am, were used to synthesize amphiphilic polymer PLENB via reversible addition-fragmentation chain transfer (RAFT) polymerization.

The structure and composition of PLENB were characterized by ^1^H NMR and FTIR. The ion-responsive characteristics of PLENB were characterized by the changes of LCST in solution under different ions and ion concentrations. The polymer micelles were prepared using a solvent evaporation induced self-assembly method. The critical micelle concentration (CMC), the morphology and distribution of the polymer micelles were studied. Finally, the application of polymer PLENB in drug delivery represented by hydrophilic gold nanoparticle and lipophilic DOX drug was further discussed.

## 2. Materials and Methods

### 2.1. Materials

LAMA was synthesized with LA (99%, Aladdin, Shanghai, China) as reported in the previous work [33,34]. Polyethylene glycol methyl ether methacrylate (PEGMA, 475 g∙mol^−1^, 97%, Aladdin) was purified by alkaline alumina column before use. *N*-isopropylacrylamide (NIPAM, 98%, Aladdin) and 2,2′-azoisobutyronitrile (AIBN) were recrystallized from *n*-hexane and ethanol, respectively. Methylacryloyl chloride (98%, J&K, Beijing, China), cumyl dithiobenzoate (CDB, Aladdin), 4-nitrobenzo-18-crown-6 (97%, Aladdin), Pd/C (75%, Aladdin), HAuCl_4_·3H_2_O (Aladdin), doxorubicin hydrochloride (Aladdin), dichloromethane, ethanol and other solvents (AR, Sinopharm Chemical Reagent Co., Shanghai, China) were not further purified.

### 2.2. Characterization

^1^H NMR spectra were recorded on Bruker Avance 400 MHz NMR spectrometer (BRUKER, Berlin, Germany) at 25 °C using DMSO as solvent. IR spectra were characterized by a Nicolet iS10 FTIR (Thermo Fisher, Waltham, MA, USA). The LCST of polymer was obtained from the transmittance examined by a UV-2550 spectrometer (SHIMADZU, Kyoto, Japan). The CMC and the drug-release were determined by FLS920 fluorescence spectrophotometer (Edinburgh Instruments Co., Livingston, UK). The morphology and distribution of polymer micelles were characterized by JEM2100 high-resolution TEM (JEOL Ltd., Tokyo, Japan) and Zetasizer NanoZS DLS (Malvern, Worcestershire, UK), respectively.

### 2.3. Synthesis of Monomer and Polymer

#### 2.3.1. Synthesis of Monomer B18C6Am

**Scheme 1:**
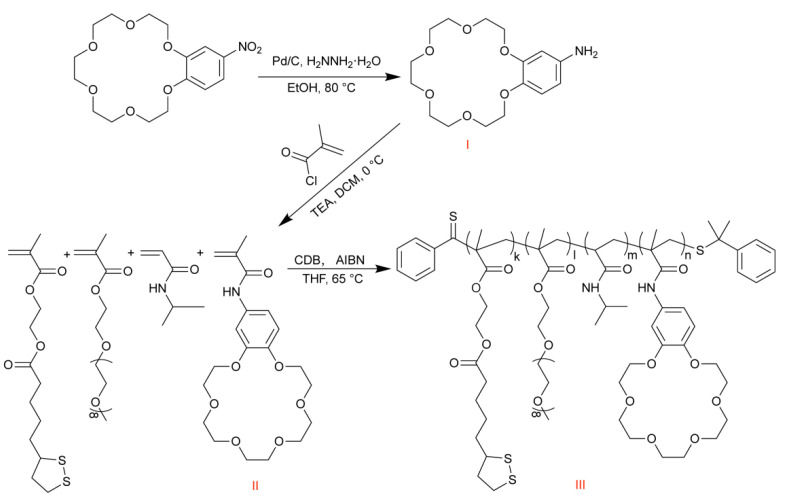
Synthesis route of (**I**) aminobenzo-18-crown-6-ether, (**II**) B18C6Am and (**III**) PLENB.

The monomer B18C6Am [35,36] was synthesized via two steps, including the reduction reaction and the amidation reaction, which was shown in Scheme 1. First, 4-nitrobenzo-18-crown-6-ether was reduced to 4-aminobenzo-18-crown-6-ether according to the following procedure. 4-Nitrobenzo-18-crown-6-ether (1 g, 28 mmol) was dissolved in ethanol at 80 °C, followed by the addition of Pd/C. The mixture of ethanol and hydrazine hydrate was added dropwise to the solution as slowly as possible, and then the reaction continued for another 12 h.

After that, Pd/C and ethanol were removed from the solution. The mixture was dissolved in DCM and extracted with deionized water to obtain 4-aminobenzo-18-crown-6-ether. Secondly, the monomer B18C6Am was obtained by the amidation reaction between 4-aminobenzo-18-crown-6-ether and methacryloyl chloride. The weighted 4-aminobenzo-18-crown-6-ether (1 g, 3.06 mmol) was dissolved in DCM at 0 °C, followed by the addition of triethylamine (850 μL, 6.12 mmol). Next, methacryloyl chloride (592 μL, 6.12 mmol) was slowly added dropwise to the above mixture, and the whole process was kept at 0 °C, and then reacted at room temperature for another 10 h. After extracting the reacted mixed solution with deionized water, it was dried, concentrated and precipitated with hexane to obtain a white powder.

4-Aminobenzo-18-crown-6-ether: FTIR (KBr, cm^−1^): 3583, 3433, 2868, 1616, 1516, 1230, 1125. ^1^H NMR (DMSO, δ/ppm, TMS): 6.64, 6.23, 6.07 (benzene ring, –C_6_H_3_–NH_2_); 4.68 (–C_6_H_3_–NH_2_); 3.98–3.89 (crown ether ring, –C_6_H_3_–O–CH_2_CH_2_–O–), 3.76–3.67 (crown ether ring, –C_6_H_3_–O–CH_2_CH_2_–O–), 3.62–3.52 (crown ether ring, –C_6_H_3_–O–CH_2_CH_2_–O–CH_2_CH_2_–O–CH_2_CH_2_–O–).

B18C6Am: FTIR (KBr, cm^−1^): 3583, 3282, 2868, 1654, 1616, 1516, 1410, 1230, 1125. ^1^H NMR (DMSO, δ/ppm, TMS): 9.58 (–CO–NH–); 7.35, 7.21, 6.89 (benzene ring, –C_6_H_3_–NH–CO–); 5.77, 5.48 (CH_2_=CH–); 4.10–3.97 (crown ether ring, –C_6_H_3_–O–CH_2_CH_2_–O–), 3.8–3.72 (crown ether ring, –C_6_H_3_–O–CH_2_CH_2_–O–), 3.65–3.52 (crown ether ring, –C_6_H_3_–O–CH_2_CH_2_–O–CH_2_CH_2_-O–CH_2_CH_2_–O–).

#### 2.3.2. Synthesis of Polymer PLENB

A certain dose of B18C6Am, NIPAM, LAMA, PEGMA, CDB and AIBN was dissolved using THF in a Schlenk tube (Chongqing Synthware Glass, Shanghai, China). The solution was deoxygenated by freezing and thawing and then polymerized at 65 °C for 48 h. After that, the mixture was precipitated with ethyl ether to obtain the target product of poly(LAMA-PEGMA-NIPAM-B18C6Am) (abbrev. PLENB). Here, three amphiphilic random copolymers with different crown ether contents were prepared by changing the feed ratio of monomers as shown in Table 1, which was labelled as PLENB1 (10%), PLENB2 (15%) and PLENB3 (30%).

### 2.4. The Lowest Critical Solution Temperature LCST Detection

#### 2.4.1. PLENB Solution without Additional Ions

When the temperature of polymer solution was near LCST, its solubility changes significantly. To obtain the LCST of PLENB in H_2_O, the 0.5 wt% aqueous solution of PLENB1, PLENB2, and PLENB3 were prepared. The three samples were placed in a water bath environment to ensure a uniform and stable temperature change of the polymer solution. Starting from room temperature, the test can be conducted every time when the temperature raises 1 °C and stabilized for 15 min. Then, we increased the temperature of the polymer solution gradually and recorded the change of transmittance. We repeated this three times for each group and calculated the average value of the three tests.

Furthermore, a certain concentration of HCl was added to the solution to adjust the pH to 4.0, and then the LCST of PLENB was also determined in the same way.

#### 2.4.2. PLENB Aqueous Solution with Additional Ions

We prepared a 150 mM KCl, NaCl and MgCl_2_ solution and their mixed solution in advance. Then, PLENB was added to the solution, and the change of LCST was detected. In addition, the change trend of LCST in the condition of different K^+^ concentrations was also explored. A series of KCl solutions was set to 0, 20, 40, 80, 100, 120, 150, 180 and 200 mM.

### 2.5. The Self-Assembly of PLENB

The PLENB polymer micelles were prepared by the solvent evaporation induced self-assembly method. We added 2 mL 0.5 mg/mL PLENB/THF solution dropwise into 10 mL deionized water. The mixture was stirred overnight without sealing. After THF was completely evaporated, the PLENB polymer micelles were formed. The morphology and the size distribution of polymer micelles were characterized by TEM and DLS. In addition, the K^+^ sensitivity of PLENB micelle could be obtained by analysing the micelle size of PLENB micelles/K^+^ system after adding a certain of 150 mM KCl to PLENB micelles and stirring overnight.

The critical micelle concentration was measured on a fluorescence spectrophotometer, using 0.02 mg/mL pyrene as a fluorescent probe. A series of PLENB micelles were prepared with different concentrations of 0.005, 0.010, 0.025, 0.05, 0.1, 0.25, 0.5 and 1.00 mg/mL. The excitation wavelength was 335 nm, and the excitation and emission slit width was 1.5 nm.

### 2.6. Drug Release Behaviour of PLENB

As for a hydrophilic system, we chose gold nanoparticles (AuNPs) as a representative. AuNPs with an average particle size of about 8 nm were prepared by the method of reducing HAuCl_4_·3H_2_O with NaBH_4_. The disulphide bond in PLENB was reduced to a sulfhydryl group by NaBH_4_ reduction method to obtain PLENB-SH. We prepared 5 mL of the AuNPs aqueous solution (0.184 × 10^−8^ mM), followed by adding 2 mL of the reduced PLENB-SH aqueous solution (0.02 g/mL).

It reacted at room temperature for 48 h to obtain PLENB-SH@Au. Then, KCl was added into the PLENB-SH@Au solution to form PLENB-SH@Au/KCl system. KCl was added at a series of concentrations ranging from 0 to 225 mM, increasing by 15 mM each time. UV-vis spectroscopy and TEM were used to detect the absorption intensity and morphology of PLENB-SH@Au/KCl system, respectively.

As for a hydrophobic system, we chose DOX as a representative. The mixture of 1.6 mg DOX, 20 μL TEA and DMSO were stirred in a dark environment until dissolution. We mixed 8 mg of PLENB3 together and stirred for another 1 h. The mixture was added dropwise to deionized water and stirred for 1 h and, finally, was transferred to a dialysis bag and dialyzed for 48 h. PLENB3 could encapsulate DOX to form PLENB3@DOX micelles and left in dialysis bag, while the free DOX would be discharged due to its small molecular weight.

After dialysis, the K^+^ triggered DOX release test was carried out. First, the dialyzed solution was divided into several equal parts, and one of them was used as control sample. Secondly, 5, 50, 100, 150 and 200 mM KCl were added into each dialyzed sample. Finally, the fluorescence spectroscopy was used to detect the release behaviour of PLENB3@DOX at 25 and 37 °C. The excitation wavelength was set to 480 nm, and the time was about 50 h.

### 2.7. Data Analysis

The molecular weight of copolymer PLENB was calculated by ^1^H NMR spectra. First, according to the integration area of 6.89 ppm (one proton of benzene ring in B18C6Am block), 4.02 ppm (two protons of –COO–CH_2_− in LAMA block), 3.79 ppm (one proton of methine in NIPAM block) and 3.24 ppm (two protons of ethoxy in PEGMA block), the block ratio of the amphiphilic copolymer PLENB was obtained. Secondly, based on the used amount of chain transfer agent CDB, the polymerization degree of PLENB was acquired and then the molecular weight of PLENB could be calculated.

As for as the data of drug loading (DLC) and encapsulation efficiency (EE), they were defined and calculated according to the follow formula [37]:

(1) $$\mathrm{DLC}\%=\frac{Weight\;of\;drug\;in\;micelle}{Weight\;of\;copolymer}\times 100\%$$

(2) $$\mathrm{EE}\%=\frac{Weight\;of\;drug\;in\;micelle}{Weight\;of\;drug\;in\;feeding}\times 100\%$$

## 3. Results

### 3.1. Characterization of Amphiphilic Polymer PLENB

**Figure 1:**
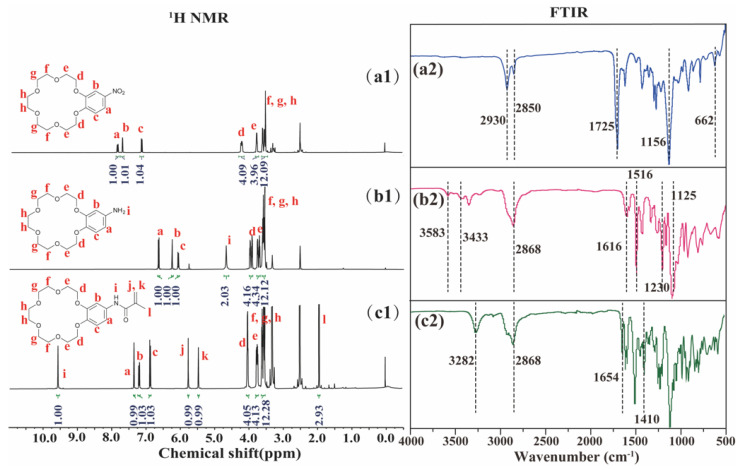
^1^H NMR and FTIR spectra of monomers: 4−nitrobenzo−18−crown-6 (**a1**,**a2**); 4−aminobenzo−18−crown−6 (**b1**,**b2**) and B18C6Am (**c1**,**c2**).

Figure 1 showed the ^1^H NMR spectra and FTIR spectra of 4-nitrobenzo-18-crown-6-ether Figure 1(a1,a2), 4-aminobenzo-18-crown-6-ether Figure 1(b1,b2) and B18C6Am Figure 1(c1,c2). It was observed that the chemical shifts of the protons (H_a_, H_b_, H_c_) on the aromatic ring structure in Figure 1(a1) moved from 7.90, 7.74 and 7.19 ppm to 6.64, 6.23 and 6.07 ppm in Figure 1(b1), respectively. At the same time, the proton peak of the amino group (H_i_) appears at 4.68 ppm in Figure 1(b1), which was assigned to 4-aminobenzo-18-crown-6-ether.

Furthermore, it can be seen from Figure 1(c1) that the amino proton peak disappeared, the proton peak on the aromatic ring moved to 7.35, 7.22 and 6.89 ppm, while the amide proton (H_i_) and the C=C double bond protons (H_j_, H_k_) appeared at the peaks of 9.58, 5.76 and 5.45 ppm, respectively. The NMR results verified that B18C6Am was successfully synthesized. Compared with the IR spectrum of 4-nitrobenzo-18-crown-6 (Figure 1(a2)), new peaks appeared in the IR spectrum of 4-aminobenzo-18-crown-6 (Figure 1(b2)).

The NH stretching vibration absorption peaks appeared at 3583 and 3433 cm^−1^, and the NH bending vibration absorption peak appeared at 1616 cm^−1^. Figure 1(c2) was the infrared spectrum of B18C6Am. It was found that the NH stretching vibration absorption peaks at 3583 and 3433 cm^−1^ disappeared, while a new stretching vibration peak of NH in amide appeared at 3282 cm^−1^.

The peak at 1516 cm^−1^ is the skeleton vibration peak of the C=C double bond on the benzene ring, the peak at 1230 cm^−1^ is the characteristic absorption of C–O linked to the benzene ring, and the peak at 1125 cm^−1^ is the C–O–C stretching vibration peak on the crown ether ring. The stretching vibration peak of the C=O bond in the amide appeared at 1655 cm^−1^, and the stretching vibration absorption peak of the CN bond of the secondary amine appeared at 1410 cm^−1^, further supporting the successful synthesis of B18C6Am.

**Figure 2:**
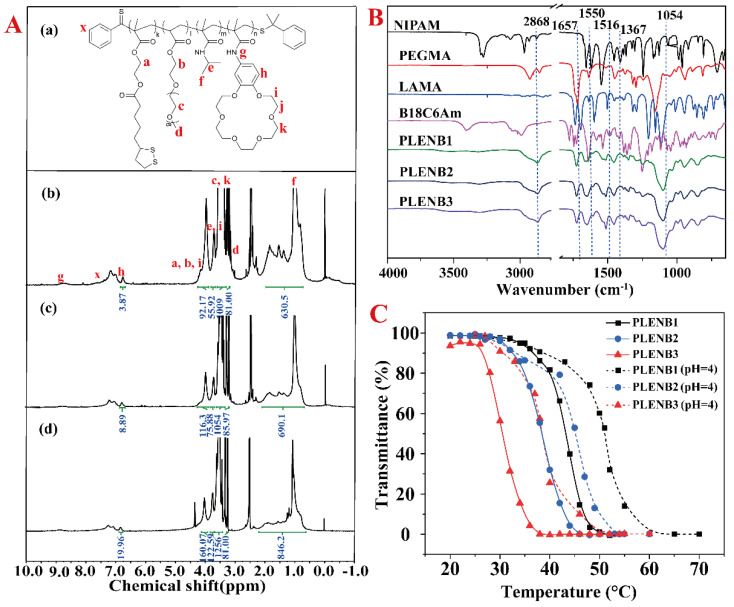
The ^1^H NMR spectra (**A**) ((**a**): the structure of PLENB; ^1^H NMR spectra of PLENB1 (**b**), PLENB2 (**c**) and PLENB3 (**d**)) and FTIR spectra (**B**) of PLENB polymer; and the LCST of PLENB polymer in both neutral and acidic environments by UV−vis spectroscopy (**C**).

Figure 2A showed the ^1^H NMR spectra of PLENB. There was no signal in the range of 5.3–5.7 ppm, indicating that the monomers had reacted completely, and there was no C=C double bond proton. The signal at 7.0–8.0 ppm belonged to the amide proton of NIPAM, and the signal at 6.89 ppm belonged to the benzene ring of B18C6Am. The ester groups of PEGMA, LAMA and the crown ether ring proton peak of B18C6Am appeared at 4.03 ppm. In addition, the signals at 3.79 ppm were assigned to the isopropyl proton of NIPAM and the crown ether ring proton of B18C6Am. This suggested that PLENB was synthesized successfully.

As the crown ether group could be adsorbed on the chromatographic column, the polymer was difficult to elute to obtain an accurate molecular weight. Therefore, the molecular weight was calculated by ^1^H NMR in this paper. According to the integration area of the characteristic peaks of B18C6Am, LAMA, NIPAM and PEGMA, the monomer molar ratio of the amphiphilic copolymer PLENB was calculated. The calculation data of PLENB with different polymerization degree and the corresponding molecular weight are shown in Table 1.

Figure 2B shows the FTIR spectra of the monomers and polymer PLENB with different polymerization degrees. In the IR spectra of PLENB, the C=O bond stretching vibration peak, the NH deformation vibration absorption peak and the CH stretching vibration peak in isopropyl group of NIPAM appeared at 1657, 1550 and 1367 cm^−1^, respectively. The peak at 1054 cm^−1^ was assigned to the characteristic absorption of C–O bond in MAPEG. The characteristic peak of C–S bond in LAMA appeared at 662 cm^−1^. In addition, the stretching vibration peak of the C=C bond on the benzene ring appeared at 1516 cm^−1^, which was the characteristic peak of B18C6Am. The FTIR results agreed with the NMR analysis, which confirmed that PLENB was synthesized successfully.

As shown in Figure 2C, the UV-vis transmittance of the polymer PLENB1 solution (pH 7.0) was higher than 75%, but when the temperature was up to 43 °C, the transmittance dropped to 39% sharply, and the transition temperature was LCST_1_. Similarly, the LCST_2_ of PLENB2 was 37 °C, and LCST_3_ of PLENB3 was 31 °C in a neutral environment. Apparently, with the increase of the B18C6Am content, the LCST of the polymer PLENB decreased gradually.

Furthermore, compared with the three curves in Figure 2C, the transmittance of the PLENB1 curve decreased slowly before reaching LCST_1_, while the decreasing rate of the PLENB2 and PLENB3 curves gradually increased with the increase of B18C6Am monomer content. As the crown ether groups increased, the ring volume effect caused space hindrance, which would partly affect the contact space probability between the NIPAM chain segment and water.

This was equivalent to reducing the total number of hydrogen bonds so that PLENB3 had the lowest LCST. In addition, B18C6Am was a hydrophobic monomer, and when other polymer conditions remained unchanged, the more hydrophobic segments in the copolymer, the more hydrophilicity of the copolymer decreased; thus, the LCST varied with the content of B18C6Am. In general, the LCST of PLENB was closely related to the B18C6Am content in the polymer.

Furthermore, the pH was adjusted to be 4.0, and the LCST was also tested. The results (Figure 2C) showed that the LCST of PLENB in acidic environment had a significant increasing compared to that in neutral environment. The LCST of PLEB1, PLENB2 and PLENB3 in pH 4.0 was 51, 45 and 38 °C, respectively. It was inferred that the pH sensitivity might be attributed to the protonation of the NIPAM-containing block.

### 3.2. LCST of PLENB in Aqueous Solution with Ion

**Figure 3:**
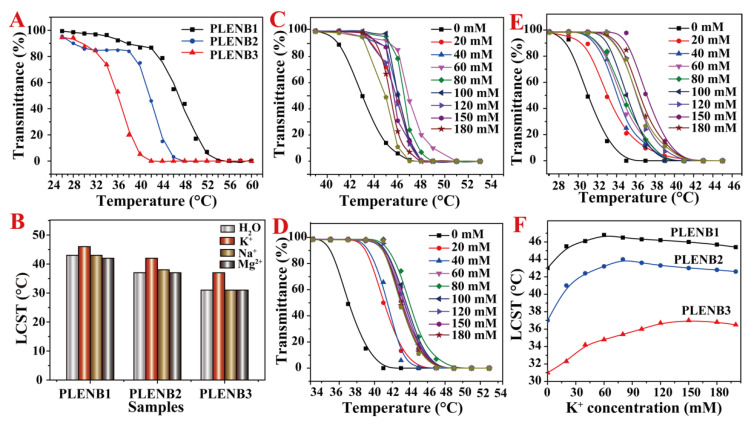
The UV−vis transmittance curves of PLENB changed with temperature in KCl solution (**A**); the LCST of PLENB in different ion solution (**B**); the transmittance curves of PLENB1 (**C**), PLENB2 (**D**), PLENB3 (**E**), and the LCST curves (**F**) of PLENB with different K^+^ concentration in different temperature.

The UV-vis transmittance curves of PLENB changed with temperature in KCl solution were shown in Figure 3A. It was clear that the LCST of PLENB changed significantly: LCST_1K_ of PLENB1 = 46 °C; LCST_2K_ of PLENB2 = 42 °C; and LCST_3K_ of PLENB3 = 37 °C. Before reaching the LCST, the transmittances of the three polymers decreased slightly. The early decline was due to the combination of KCl and surrounding water molecules in the initial stage, resulting in partial precipitation of PLENB and a decrease in transmittance.

Subsequently, we found that the LCST of PLENB1, PLENB2 and PLENB3 increased by 3, 5 and 6 °C, respectively. This was because the B18C6Am coordinated with K^+^, thereby, improving the hydrophilicity of PLENB and increasing the hydrogen bonding force. The higher the crown ether content, the stronger the hydrogen bonding force, and the higher temperature required to break the hydrogen bond, the higher the LCST.

**Scheme 2:**
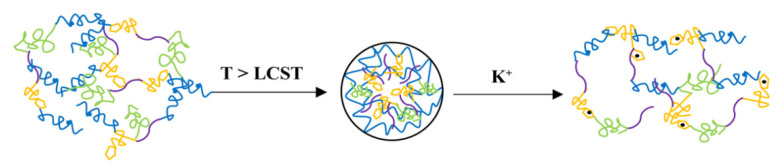
Schematic diagram of the conformational changes of PLENB in solution.

As shown in Scheme 2, when the environment temperature was higher than LCST, the conformation of polymer shrunk from random coil to spheroid. When KCl was added, the crown ether coordinated with K^+^ and the conformation transformed into random coils again. Therefore, the existence of K^+^ played an important role in the process of the conformation transformation and the LCST improvement.

The ion responsiveness of Na^+^ and Mg^2+^ were further explored by probing the changes of LCST. Figure 3B showed the bar graph of PLENB′s LCST in different ions. Clearly, the LCST of all PLENB had a significant increase in K^+^ solution, while there was only a slight change in the Na^+^ and Mg^2+^ solutions. The slight fluctuation of the LCST could be explained by inorganic salt competitively binding with water molecules to weaken the solubility of polymer.

In summary, the prepared crown ether-based amphiphilic random copolymer PLENB was only selective for K^+^. Since the synthesized PLENB was sensitive to K^+^ rather than other ions, we further discussed the influence of K^+^ concentration on LCST. Figure 3B–D were the curves of PLENB1 (C), PLENB2 (D) and PLENB3 (E) transmittance, and the LCST curves (F) of PLENB varied with K^+^ concentration. It can be seen that the LCST of PLENB increased gradually with the increase of the K^+^ concentration.

However, when the ion concentration reached a certain value, the LCST reached the maximum value. The LCST of PLENB1, PLENB2 and PLENB3 reached the maximum at K^+^ concentrations of 60, 90 and 150 mM, respectively. After that, LCST decreased with the increase of K^+^ concentration. This trend is more directly reflected in Figure 3F. The molar weight of crown ethers in PLENB1, PLENB2 and PLENB3 were 0.0106, 0.0192 and 0.0363 mmol, while the K^+^ molar weight at the turning points were 0.6, 0.9 and 1.5 mmol, respectively.

When the molar ratio of K^+^ to crown ether was about 52.5, 47.4 and 42.1, the LCST of PLENB1, PLENB2 and PLENB3 reached their maximum values, respectively. When the concentration of K^+^ was 40–50-times as much as the number of crown ether units, the LCST of PLENB reached its maximum value. If the concentration of K^+^ was further increased, the excess salt in the solution would bind water molecules around the polymer competitively, which made the polymer easier to precipitate, and LCST gradually decreased.

On the one hand, the addition of K^+^ would coordinate with crown ethers. On the other hand, it may destroy the hydrogen bond. When K^+^ mainly broke hydrogen bonds, the LCST of polymer decreased, while when K^+^ mainly coordinated with crown ether, the LCST increased. We concluded that the influence effect of K^+^ on LCST was complex. The final effect depended on the molar ratio of K^+^ to crown ether in polymer.

Therefore, the rising or falling trend usually appeared in the initial stage or later stage. Here, the LCST appeared increased first and then decreased. The number of crown ether in the polymers was enough, and K^+^ mainly coordinated with crown ethers. Therefore, the higher the concentration of K^+^ was, the higher the LCST was. When the crown ether was saturated by the coordinating with K^+^, the excess K^+^ mainly broke the hydrogen bonds, and the LCST of the polymer gradually decreased.

### 3.3. Self-Assembly of Amphiphilic Copolymer PLENB

**Figure 4:**
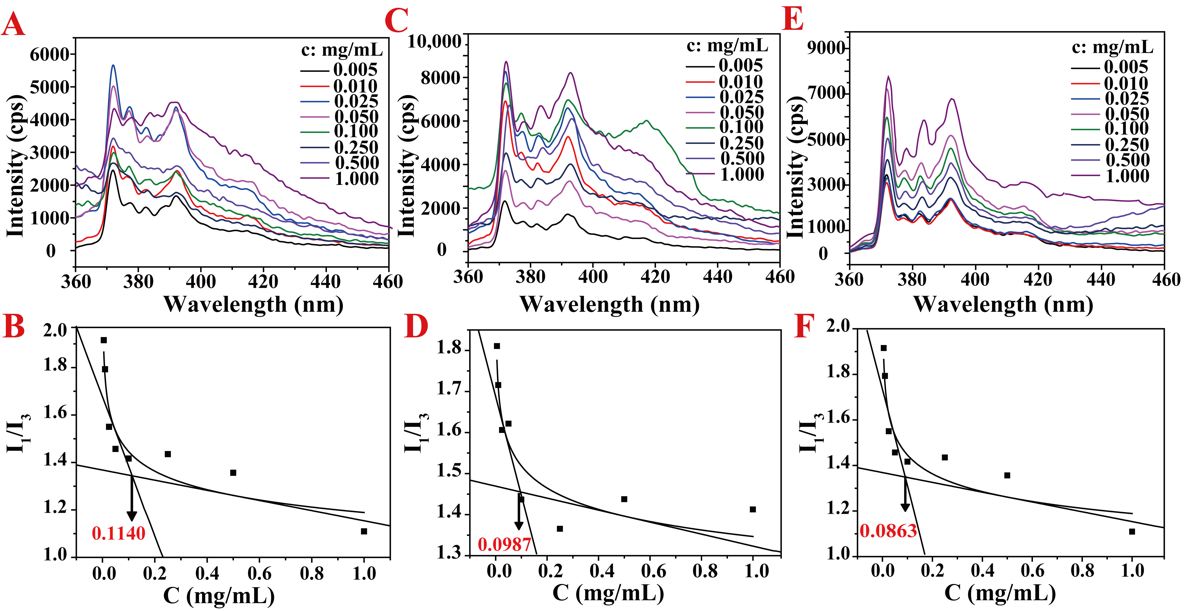
Fluorescence emission spectra (**A**,**C**,**E**) and the relative curve of I_1_/I_3_ vs. concentration (**B**,**D**,**F**) of pyrene in PLENB solution. (**A**,**B**) PLENB1, (**C**,**D**) PLENB2, (**E**,**F**) PLENB3.

As for the pyrene molecule, the fluorescence emission wavelengths of single molecule and the excimer were 373 nm (I_1_) and 383 nm (I_3_), respectively. The I_1_/I_3_ value represents the polarity of the system, and it can drop sharply when reaching the critical micelle concentration [38]. It can be seen from Figure 4A that, when the PLENB1 had a low concentration, I_1_ (2476 cps) was higher than I_3_ (1684 cps). As the concentration of PLENB1 gradually increased, I_3_ gradually increased.

The specific fluorescence curves of PLENB2 (C) and PLENB3 (E) polymer solutions also showed a similar trend to PLENB1. Based on this principle, the relationship between I_1_/I_3_ and polymer concentration in the pyrene fluorescence spectra was established. As shown in Figure 4B, the value of I_1_/I_3_ decreased significantly as the concentration of PLENB1 increased. The decline rate became slower when the concentration of PLENB1 reached 0.1140 mg/mL, which was called the turning point.

The measured CMC of PLENB1 was 0.1140 mg/mL. At this moment, the micelle was formed, then the solubilization of pyrene was improved and more excimer complexes appeared, which caused the value of I_1_/I_3_ to decrease. Similarly, in Figure 4D,F, the CMC of PLENB2 and PLENB3 were measured to be 0.0987 and 0.0863 mg/mL, respectively.

The CMC of amphiphilic copolymer generally increased with the increase of the hydrophilic segment and decreased with the increase of the hydrophobic segment. This was because, when the number of hydrophilic segments increased, the strong interaction between the polymer and water molecules was difficult to form micelles, and when the number of hydrophobic segments increased, the hydrophobic segments promoted molecular self-assembly and aggregation.

**Figure 5:**
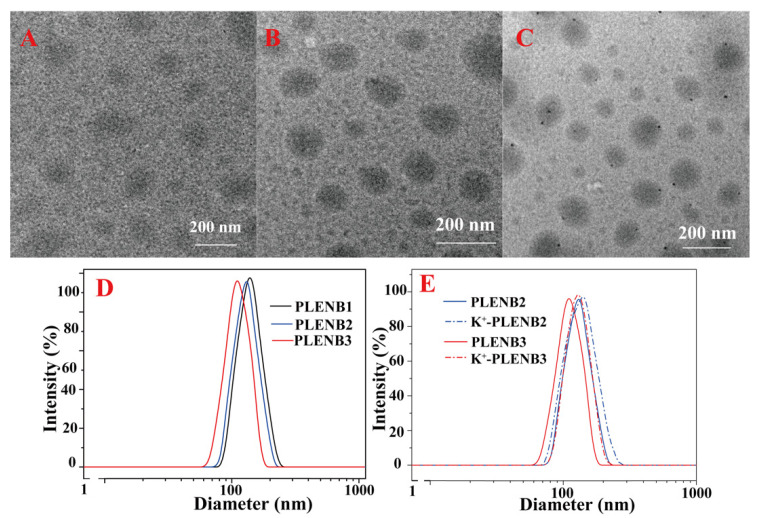
TEM diagram of (**A**) PLENB1, (**B**) PLENB2, (**C**) PLENB3 micelles, and DLS of (**D**) PLENB micelles, (**E**) PLENB micelles/K^+^ solution.

TEM and DLS were conducted to characterize the morphology and particle size distribution of the PLENB1, PLENB2 and PLENB3 micelles. It can be seen from Figure 5 that the micelles of PLENB1 (A), PLENB2 (B), and PLENB3 (C) were all small spherical structure with good dispersion. The calculated sizes of PLENB1, PLENB2 and PLENB3 micelles were 134 ± 3, 120 ± 5 and 95 ± 2 nm, respectively. Figure 5D shows the DLS diagram of PLENB micelles.

Three curves presented single peak and narrow shape, indicating that the micelles had relatively uniform particle size. Next, we took an appropriate amount of the above PLENB2 and PLENB3 micelles to mix with 150 mM KCl. The DLS characterization of the mixture is shown in Figure 5E. It can be seen that, in K^+^ solution, the particle size of PLENB2 micelles increased from 130 to 140 nm, whereas that of PLENB3 micelles increased from 110 to 130 nm.

This might be explained by the reason that the coordination of K^+^ with the crown ether in the polymer micelles increased the hydrophilicity of the PLENB micelles/K^+^ causing the PLENB micelles more stretchable in solution. We found that the particle size of PLENB2 micelles increased by about 10 nm, while that of PLENB3 increased by about 20 nm. This difference was consistent with the fact that PLENB2 and PLENB2 contained different amounts of crown ethers. The repeated unit numbers of crown ethers in PLENB2 and PLENB3 were 9 and 20, respectively. Therefore, compared with PLENB2, PLENB3 was more likely to interact with K^+^, and then increased its micelle size to a greater extent.

### 3.4. K^+^-Triggered Disassembly of PLENB3-SH@Au Aggregates

**Figure 6:**
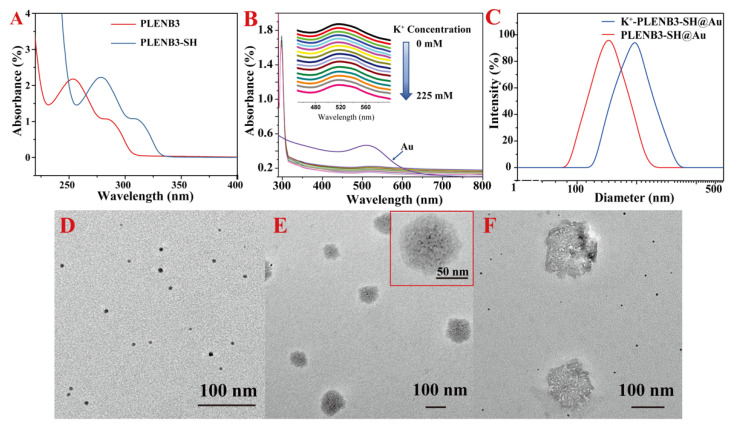
(**A**) UV−vis spectra of PLENB3 and PLENB3-SH; (**B**) UV−vis spectra of Au NPs, PLENB3-SH@Au, and K^+^-PLENB3-SH@Au with different K^+^ concentration; (**C**) DLS of PLENB3-SH@Au and K^+^-PLENB3-SH@Au; (**D**) TEM image of AuNPs; (**E**) TEM images of PLENB3-SH@Au, and the inset was the magnified view; (**F**) TEM image of K^+^-PLENB3-SH@Au.

It is worth exploring the assembly and disassembly of Au aggregates because Au NPs have many excellent properties, especially in the field of catalysis and biology [39,40]. We attempted to use PLENB-SH as a ligand to combine with Au NPs to achieve the assemblies. Inversely, we used the K^+^ ion to induce disassembly. We noticed that PLENB with the disulphide bond was reduced to PLENB3-SH containing dithiol before using. Figure 6A shows the UV-vis spectra of PLENB and PLENB3-SH. The absorption band caused by the π−π transition on the benzene ring of PLENB3 appeared at 283 nm, which moved to 304 nm in PLENB-SH.

The C=O absorption peak at 253 nm in PLENB3 shifted to 279 nm in PLENB3-SH. As shown in Figure 6B, the absorption peak of Au NPs was located at 510 nm. With the addition of PLENB3-SH, the absorption peak shifted to 518 nm because of the effect of Au-S coordination, which meant the formation of PLENB3-SH@Au. However, minor changes were observed in the UV-vis spectra when K^+^ continued to be gradually added to the PLENB3-SH@Au solution. In order to further ascertain the influence of K^+^ on PLENB3-SH@Au aggregates, DLS and TEM were used to measure their size and morphology. Figure 6C shows the DLS curves of PLENB3-SH@Au and K^+^-PLENB3-SH@Au.

It can be seen that the hydrodynamic size of K^+^-PLENB3-SH@Au was about 195 nm, which was larger than that of PLENB3-SH@Au. The reason was that the presence of K^+^ caused the coordination with crown ether and then improved the hydrophilicity of PLENB-SH polymer chain, resulting in a bigger swelling degree and a looser aggregation structure. If the hydrophilicity of PLENB-SH in the PLENB-SH@Au assemblies reached an appropriate level, the Au NPs might be separated from the K^+^-PLENB3-SH@Au aggregates. This was further confirmed by TEM results.

Figure 6D shows that the size of original Au NPs was about 8 ± 0.5 nm with a regular morphology. The TEM image of PLENB3-SH@Au (Figure 6E) shows that Au NPs loaded on the PLENB3-SH micelles, and the size of PLENB3-SH@Au aggregates were about 105 ± 5 nm. Figure 6F is a TEM image of PLENB3-SH@Au in the presence of K^+^ with a concentration of 150 mM. It can be seen that Au NPs disassembled from the micelles, whereas, the size of K^+^-PLENB3-SH micelles increased to 150 ± 6 nm. In summary, Au NPs were disassembled from the PLENB3-SH@Au aggregates due to the loose structure of K^+^-PLENB3-SH.

### 3.5. K^+^-Triggered Release of DOX

**Figure 7:**
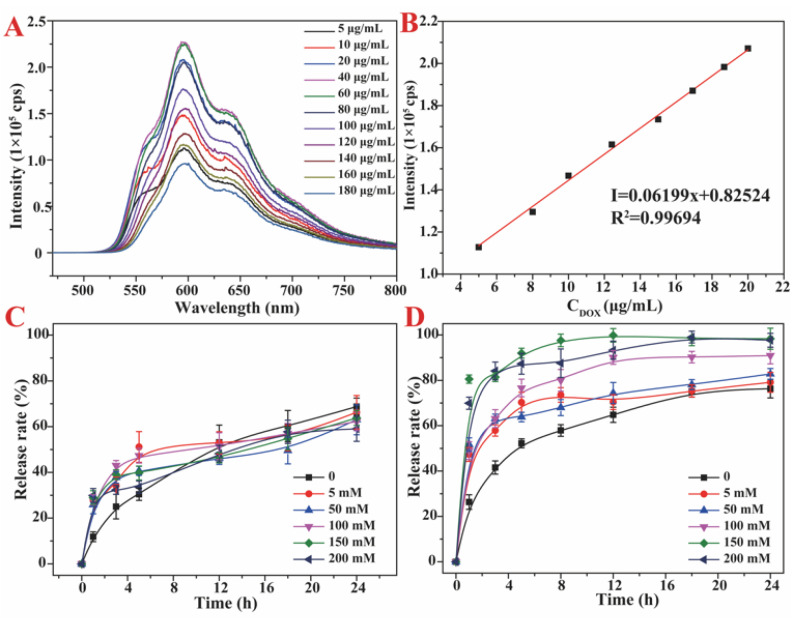
The fluorescence vs. concentration curves of DOX (**A**) and its standard fluorescence curve (**B**), release curves of PLENB3 drug-loaded micelles under different K^+^ concentrations and different environmental temperature: room temperature (**C**) and 37 °C (**D**).

As shown in Figure 7A,B, the standard fluorescence curve was obtained by plotting the DOX fluorescence curve at different concentrations and the linear equation was I = 0.6199x + 0.82524 and R^2^ = 0.99694. According to the calculation formula, we concluded that the DLC of PLENB3 was 7.67%, and the EE was 39.01%. The obtained drug-loaded micelles were divided into several, and their drug release behaviours at different K^+^ concentrations and different environmental temperatures were monitored in real time.

Figure 7C,D show the release rate of DOX at room temperature and 37 °C within 24 h. It was clear that the release behaviour of DOX in PLENB3 micelles at room temperature was less affected by the K^+^ concentration in Figure 7C. Within 24 h, the release rate of DOX in micelle solution without K^+^ was 68.77% at room temperature, while that of the solution with 150 mM K^+^ was 63.78%. At room temperature, adding K^+^ did not increase the release rate of DOX. This indicated that the combination of K^+^ and crown ether made the LCST of polymer micelles increase.

Polymer micelles were more stable, and DOX release became slower. In Figure 7D, the release behaviour of DOX in PLENB3 micelles is closely related to the K^+^ concentration. At 37 °C, the release rate of DOX in the micelle solution without K^+^ was 76.33% within 24 h, while that of the micelle solution with 150 mM K^+^ reached 99.84%. The solution completed the release of drug within 12 h, which greatly sped up the drug release process.

Comparing the release behaviour of micelle solution with different K^+^ concentrations, we found that the release rate of micelles was continuously increasing with K^+^ concentration in the range of 0–150 mM, while the release rate showed a downward trend when the concentration was 200 mM. When excessive K^+^ was added, the LCST of the polymer was higher than 37 °C, which made it difficult to release DOX. However, too much excess K^+^ would compete with polymer for water molecules, which made the hydrophilicity of micelles decrease and then reduced the release of DOX.

In general, the drug release rate of PLENB3 micelles reached up to 99.84% within 12 h under the condition of 37 °C and 150 mM K^+^, which could broaden the application of PLENB polymers in the field of drug delivery.

## 4. Conclusions

The amphiphilic random polymers poly(LAMA-PEGMA-NIPAM-B18C6Am) with different crown ether contents were synthesized by designing different feeding ratios. The ratio of crown ether units in PLENB1, PLENB2 and PLENB3 were 4, 9 and 20, respectively. The corresponding molecular weights of PLENB1, PLENB2 and PLENB3 were 2.11 × 10^4^, 2.35 × 10^4^ and 2.74 × 10^4^ g/mol, respectively. In neutral aqueous solution, the LCST of PLENB1, PLENB2 and PLENB3 were 43, 37 and 31 °C, while those in acidic environments were 51, 45 and 38 °C, respectively, which indicated that PLENB was sensitive to pH.

When the PLENB copolymers were in 150 mM K^+^ solution, the LCST were increased to 46, 42 and 37 °C, respectively. The existence of K^+^ played an important role in the process of the conformation transformation of PLENB and the corresponding LCST improvement because the crown ether coordinated with K^+^, and the conformation transformed into random coils. In addition, the LCST of PLENB in Na^+^ and Mg^2+^ solution did not change significantly, confirming the characteristic responsiveness of PLENB to K^+^. We found that PLENB could self-assemble into micelles with regular morphology and uniform particle size.

In K^+^ solution, the particle size of PLENB2 micelles increased from 130 to 140 nm, and that of PLENB3 micelles increased from 110 to 130 nm. This could be explained as the coordination of K^+^ with the crown ether in the polymer micelles increased the hydrophilicity of the PLENB micelles/K^+^ and made the PLENB micelles more stretchable in solution. Furthermore, the size of aggregates increased from 105 ± 5 to 150 ± 6 nm in the presence of K^+^. The presence of K^+^ promoted PLENB3-SH@Au composited micelle swelling and made the polymer chains and polymer micelles loose, which let gold nanoparticles disassemble from the PLENB3-SH@Au aggregates.

The addition of K^+^ accelerated the release of DOX from PLENB micelles. The DOX release rate of PLENB3 micelles reached to 99.84% within 12 h under the conditions of 37 °C and 150 mM K^+^. Overall, this work is helpful to develop the crown ether-based amphiphilic polymer with more functionality for better application.

## Figures and Tables

**Table 1:** The calculation molecular weight of PLENB with different feed ratios.

Sample	LAMA:PEGMA:NIPAM:B_18_C_6_Am		Molecular Weight (g/mol)
	The Ratio of Monomers in ^1^H NMR	The Polymerization Degree	
PLENB1	1.5:6.78:10.28:1	6:27:41:4	2.11 × 10^4^
PLENB2	1.0:4.5:7.0:1.5	6:28:42:9	2.35 × 10^4^
PLENB3	0.3:1.35:2.1:1.0	6:27:42:20	2.74 × 10^4^

## Data Availability

Data available in a publicly accessible repository.
